# The Effect of Breathing Patterns Common to Competitive Swimming on Gas Exchange and Muscle Deoxygenation During Heavy-Intensity Fartlek Exercise

**DOI:** 10.3389/fphys.2021.723951

**Published:** 2021-11-24

**Authors:** Kevin J. Grossman, David J. Lim, Juan M. Murias, Glen R. Belfry

**Affiliations:** ^1^School of Kinesiology, The University of Western Ontario, London, ON, Canada; ^2^Faculty of Kinesiology, University of Calgary, Calgary, ON, Canada

**Keywords:** apnea, regulated breathing, gas exchange, muscle deoxygenation, swimming, front crawl

## Abstract

During competitive freestyle swimming, the change of direction requires a turn followed by ∼15 m of underwater kicking at various intensities that require a ∼5 s breath-hold (BH). Upon surfacing, breathing must be regulated, as head rotation is necessary to facilitate the breath while completing the length of the pool (∼25 s). This study compared the respiratory and muscle deoxygenation responses of regulated breathing vs. free breathing, during these 25–5 s cycles. It was hypothesized that with the addition of a BH and sprint during heavy-intensity (HVY) exercise, oxygen uptake (VO_2_) and oxygen saturation (S_at_O_2_) would decrease, and muscle deoxygenation ([HHb]) and total hemoglobin ([Hb_tot_]) would increase. Ten healthy male participants (24 ± 3 years) performed 4–6 min trials of HVY cycling in the following conditions: (1) continuous free breathing (CONLD); (2) continuous with 5 s BH every 25 s (CONLD-BH); (3) Fartlek (FLK), a 5 s sprint followed by 25 s of HVY; and (4) a combined Fartlek and BH (FLK-BH). Continuous collection of VO_2_ and S_at_O_2_, [Hb_tot_], and [HHb] *via* breath-by-breath gas analysis and near-infrared spectroscopy (normalized to baseline) was performed. Breathing frequency and tidal volumes were matched between CONLD and CONLD-BH and between FLK and FLK-BH. As a result, VO_2_ was unchanged between CONLD (2.12 ± 0.35 L/min) and CONLD-BH (2.15 ± 0.42 L/min; *p* = 0.116) and between FLK (2.24 ± 0.40 L/min) and FLK-BH (2.20 ± 0.45 L/min; *p* = 0.861). S_at_O_2_ was higher in CONLD (63 ± 1.9%) than CONLD-BH (59 ± 3.3%; *p* < 0.001), but was unchanged between FLK (61 ± 2.2%) and FLK-BH (62 ± 3.1%; *p* = 0.462). Δ[Hb_tot_] is higher in CONLD (3.3 ± 1.6 μM) than CONLD-BH (-2.5 ± 1.2 μM; Δ177%; *p* < 0.001), but was unchanged between FLK (2.0 ± 1.6 μM) and FLK-BH (0.82 ± 1.4 μM; *p* = 0.979). Δ[HHb] was higher in CONLD (7.3 ± 1.8μM) than CONLD-BH (7.0 ± 2.0μM; Δ4%; *p* = 0.011) and lower in FLK (6.7 ± 1.8μM) compared to FLK-BH (8.7 ± 2.4 μM; *p* < 0.001). It is suggested that the unchanged VO_2_ between CONLD and CONLD-BH was supported by increased deoxygenation as reflected by decreased Δ[Hb_tot_] and blunted Δ[HHb], *via* apneic-driven redistribution of blood flow away from working muscles, which was reflected by the decreased S_at_O_2_. However, the preserved VO_2_ during FLK-BH vs. FLK has been underpinned by an increase in [HHb].

## Introduction

Competitive swimming requires the performance of high-intensity work while performing regular periods of apnea. For example, the swimming “flip-turn” and push-off technique, facilitating a change in direction at the end of the pool, is a maneuver that requires high-power output (PO) of the lower extremities (kicking) combined with apnea. International swimming rules stipulate that the swimmers must surface after covering a maximum of 15 m from the underwater kicking phase. This underwater kicking phase endures for ∼5 s. While the backstroke affords swimmers to breathe freely during swimming as their face is not underwater, the breaths during the front crawl, performed in the prone position, is confined to the rhythmical rotation of the body along the sagittal plane in the position to when the face is exposed to the air once every stroke cycle. This imposes a regulated breathing paradigm dictated by the specific characteristics of this swimming stroke.

Previous work by Lim et al. (2018) simulated the cardiorespiratory response of the lower extremities to multiple laps of backstroke swimming by repeating cycles of 25 s of free-breathing, the approximate duration of swimming one length of a 50 m pool, with 5 s of breath-holding (BH) as are experienced during the aforementioned flip-turn and push-off phases. However, since these trials were performed on a cycle ergometer on land, as opposed to in water with facial submersion, their observations provided only a cursory understanding of the physiological effects of the ventilatory techniques that may be common to swimmers. Some swimmers, with better underwater hydrodynamics, may choose to perform a 5 s sprint kick, in combination with the BH, with the hope that the added swimming speed will offset any negative physiological effects of the sprint in the latter stages of the race. These apneic exercise interventions were separated into four 6 min bouts on a cycle ergometer performed at an intensity corresponding to a PO of 50% of the difference between the lactate threshold (LT) and maximal oxygen consumption (VO_2max_) of an individual (Δ50%): (1) constant load (CONLD), (2) CONLD with 5 s BHs every 25 s (CONLD-BH), (3) Fartlek (25 s at Δ50% and a 5 s sprint) (FLK), and (4) FLK with 5 s BHs every 25 s (FLK-BH). The addition of a BH, which reduced breathing opportunities within CONLD (CONLD-BH), elicited an increase in minute ventilation (V_E_) during the 25 s free-breathing periods, along with the elevated deoxyhemoglobin-to-VO_2_ ratio ([HHb]/VO_2_) during the 5 s BHs. This reflected greater local muscle deoxygenation that supported O_2_ utilization and mitigated the repeated hypoxia of the 5 s BH. They suggested that the stimulus for increased ventilation was underpinned by the transient increases in the end-tidal partial pressure of CO_2_ (P_ET_CO_2_) and decreases in O_2_ (P_ET_O_2_) during the 5 s BHs, as these factors have been indicated to modulate ventilatory responses (Whipp and Davis, 1979). Similar to previous apnea studies, the observed decrease in mean concentration of total hemoglobin ([Hb_tot_]) and mean concentration of deoxyhemoglobin ([HHb]) during the 5 s apnea period of CONLD-BH compared to CONLD suggests an overall reduction in blood flow and greater reliance on O_2_ extraction to support O_2_ utilization, reflecting the previously observed apnea-driven O_2_ conservation response at the level of the muscle (Andersson et al., 2004). The BH did not impose a great enough physiological stress to cause a reduction in PO to either the CONLD-BH or FLK-BH condition. However, the added stress of the sprint resulted in decreased VO_2_, despite increases in [Hb_tot_] and [HHb]. The redistribution of blood flow and reduced cardiac output (Andersson et al., 2004) have been replicated earlier under apneic conditions (Lindholm and Linnarsson, 2002; Hoffmann et al., 2005) and are similarly experienced during deep diving (Ferrigno et al., 1997). The supply of Hb to the blood has also been observed during these dives as a function of splenic contractions (Hurford et al., 1990), which elicits greater O_2_ and CO_2_ carrying capacities in blood. This deep diving response does not appear to extend to high-intensity knee extensions (Kennedy et al., 2008) or cycling exercise (Chacaroun et al., 2019) under hypoxic conditions.

Prone swimming strokes impose a regulated breathing paradigm that should abolish the transient increases in V_E_ during free-breathing. However, to what extent this is true remains unknown and could be evaluated by imposing a paradigm where regulation of frequency of breathing (f_B_) and inspiratory tidal volume (VTI) of each individual are controlled for each minute of exercise.

Therefore, the purpose of this study was to investigate respiration and muscle deoxygenation under regulated breathing vs. free-breathing conditions between the aforementioned protocols. We hypothesized that with the reduced breathing opportunities from the regulated ventilation during the 25 s breathing periods, (1) mean VO_2_ would decrease, and mean VCO_2_ would decrease in CONLD-BH compared to CONLD and in FLK-BH compared to FLK. Moreover, (2) in comparison to the conflicting results between CONLD-BH and FLK-BH as suggested by Lim et al. (2018), we expected to observe increases in [Hb_tot_], [HHb], and [La^–^] to account for the increased physiological stress of regulated breathing during both the conditions.

## Materials and Methods

Ten males (mean 23.7 ± 2.5 years) participated in this study after their written informed consent was given. Inclusion criteria were that the participants were healthy and active (i.e., exercising one to three times per week). Smokers and individuals who take medication for the cardiopulmonary system were excluded. None of the participants were involved in sports. However, they were participating in 30–60 min of moderate- to heavy-intensity (HVY) resistance training of the upper and lower extremities two to three times per week. All procedures were approved by the Western University Research Ethics Board for Health Sciences Research Involving Human Participants and were in accordance with the 1964 Helsinki Declaration and its later amendments or comparable ethical standards. Baseline characteristics for the participants are summarized in Table 1.

**TABLE 1:** Baseline participant characteristics and results from the incremental ramp test (*n*=10).

Variable	Mean ± SD
Age	23.7 ± 2.5
Height (cm)	175 ± 6
Weight (kg)	78.5 ± 9.0
VO_2max_ (L/min)	3.23 ± 0.63
VO_2_ at LT (L/min)	1.72 ± 0.16
PO at LT (W)	155 ± 23
PO at Δ20% (W)	189 ± 23
PPO (W)	303 ± 36

*LT, lactate threshold; SD, standard deviation.*

### Test Conditions

The activity levels of participants were maintained throughout the duration of this study. Participants were asked to avoid caffeine for 6 h before each test. Five tests were performed on an electronically braked cycle ergometer, each on a separate day with a minimum interval of 48 h between the tests. Participants wore a nose clip to prevent nose breathing and a mouthpiece to facilitate gas exchange analysis and ventilatory measurements.

#### Ramp Incremental Test (Day 1)

Participants were instructed to complete a ramp test to exhaustion on a cycle ergometer. The PO was increased by 25 W/min, while a cadence of 70 rpm was maintained. Verbal encouragement was given to participants to maximize their performance. When participants were unable to cycle above 60 rpm for more than five consecutive seconds, the protocol was stopped. This incremental test was used to determine the maximal aerobic capacity (VO_2max_), peak power output (PPO), and the estimated LT. Above the LT, excess [H^+^] derived from an increase in lactate production is buffered by the carbonic anhydrase reaction, which yields greater CO_2_ output in relation to O_2_ utilization than that observed when exercising below the LT (Beaver et al., 1986). This increased CO_2_ results in an increase in V_E_, which is reflective of the LT. Therefore, this estimated LT was used as a proxy of the actual LT as the exercise intensity at which VCO_2_/VO_2_ began to increase disproportionately to increases in PO (also referred to as the gas exchange threshold).

#### Constant Load Exercise (CONLD)

A constant load step procedure (CONLD) was performed at the PO at 20% of the difference between LT and VO_2max_ (Δ20%) of an individual. A 4 min baseline period at 20 W was followed by constant load cycling at 70 rpm for 6 min while free-breathing in order to stabilize the gas exchange response. Five f_B_ measurements were recorded at 1, 2, 3, 4, and 5 min after the onset of the PO.

#### Constant Load With BHs (CONLD-BH)

Another square-wave test similar to CONLD was performed by participants at Δ20% at 70 rpm. Every 25 s from the beginning of the warm-up, participants performed a 5 s BH. A 5 s countdown was given before each BH period. During the remaining 25 s of the 30 s cycles, breathing was regulated to CONLD: participants matched their f_B_ and VTI for each minute of exercise to that achieved during the CONLD condition, with the guidance of a metronome and feedback from the attending researcher, respectively.

#### Fartlek

After the initial 4 min warm-up, participants commenced HVY work at Δ20% for 6 min with a cadence of 70 rpm. Every 25 s, a 5 s interval at the PPO of an individual was performed. Subjects could breathe freely. Like in CONLD, f_B_ measurements were recorded for each of the first 5 min after the onset of exercise.

#### Fartlek With BHs (FLK-BH)

Finally, a similar protocol to FLK was performed by participants. Notably, 5 s BHs were incorporated every 25 s, so the sprints were performed under apnea. f_B_ and Vt were matched to the minute-by-minute measurements taken in FLK.

The ramp protocol was performed always on day 1. Each participant was required to complete CONLD and FLK before CONLD-BH and FLK-BH, respectively, to establish the ventilatory thresholds for the BH protocols. Otherwise, each participant was randomly prescribed one of the six possible orders to complete the submaximal exercise conditions (e.g., CONLD, FLK, FLK-BH, and CONLD-BH) by an online random sequence generator. Comparisons of the physiological outcomes were made within the performance of each participant between apneic and non-apneic conditions, such that participants acted as their own controls.

#### Experimental Considerations

During pilot testing, we noted that no individuals were able to complete the 6 min work at the prescribed Δ50% PO with the periodic BHs and/or sprints. The PO was reduced until all participants were able to complete these 6 min trials. This PO corresponded with a prescribed PO of Δ20%.

### Measurements

Breath-by-breath gas exchanges and ventilatory rates at the mouth were assessed using a mass spectrometer (Innovision, AMIS 2000, Lindvedvej, Denmark) and are described in detail elsewhere (Babcock et al., 1994). Briefly, flow rates during inspiration and expiration were determined with a low dead space bidirectional turbine (Alpha Technologies, Laguna Beach, CA, United States, VMM 110) and pneumotach (Hans Rudolph, Shawnee, Kansas, United States, Model 4813) calibrated with a 3 L syringe. Gas samples at the mouth were analyzed for O_2_, CO_2_, and N_2_ concentrations. Changes in concentrations of gases were matched to the corresponding increase or decrease in volumes of the gases. There was a 20 ms interval between collection samples that were sent electronically to a computer to analyze individual breaths. Each breath started with inspiration and ended with expiration. Therefore, each 5 s BH was recorded as a single breath.

The procedures for near-infrared spectrometry (NIRS) data collection were similar to those previously described (Inglis et al., 2017). Continuous measurement of the quadriceps was performed with a NIRS device (Oxiplex TS, model 95,205, ISS, Champaign, IL, United States). Laser diodes pulsed quickly (110 MHz) at two different wavelengths near the infrared region (690 and 828 nm). These were connected to a plastic probe that was placed midway between the lateral epicondyle and the greater trochanter of the femur on the belly of the vastus lateralis muscle head. An elastic strap secured the device in place. An optically dense, black vinyl sheet was placed over the device to prevent the exposure to extraneous light. A tensor bandage was wrapped gently around the leg of the participant to secure the NIRS device to the site of interest and to further prevent the intrusion of external light into the site of NIRS measurement. Care was taken to ensure that no blood flow occlusion occurred to the leg. Deoxygenated hemoglobin concentration ([HHb]) and oxygenated hemoglobin ([HbO]) were measured, whereas [Hb_tot_] and tissue oxygen saturation (S_at_O_2_) were derived with this apparatus. [Hb_tot_] was calculated as the sum of [HHb] and [HbO], and S_at_O_2_ was estimated as the percentage of [HHb] to [Hb_tot_]. To account for individual differences in absolute tissue absorption, [HHb] and [Hb_tot_] were adjusted to baseline values (Δ[HHb] and Δ[Hb_tot_], respectively).

Rubbing alcohol was applied to the left index finger of each participant before each test. Blood lactate concentrations ([La^–^]) were taken 3 min pre- and post-exercise for each test. A lancet (ACCU-CHEK Safe-T-Pro Plus) exposed blood on the finger, which was examined by the SensLab GmbH Lactate SCOUT arterialized-capillary lactate analyzer.

### Analysis

Gas exchange and NIRS data were cleaned by the removal of aberrant data points that were at least 3 SDs from the local mean. The data were interpolated linearly to convert from breath-by-breath to 1 s intervals. Then, data points were averaged into 5 s bins. This analysis technique has been described previously (Keir et al., 2014). [HHb] and [Hb_tot_] were adjusted to zero with baseline as described earlier. These baselines represented the average of 60 s before the square-wave change in PO. Δ[HHb]/VO_2_ was determined as the ratio of the normalized [HHb] to VO_2_. Between-group comparisons on gas exchange and NIRS variables were performed from the start of the square-wave change in PO (the exercise on-transient) to the end of the bout (0–360 s). Within-group comparisons were performed between the first 25 s and the last 5 s of each 30 s cycle for VO_2_ and VCO_2_. Lamarra et al. (1987) described this mono-exponential function that models the on-transient VO_2_ curve during a step transition:


(1) $$y\,\left(t\right)=y_{B\,S\,L}+A_{p}\,\left(1-e^{-\left(t-TD\right)/\tau )}\right)$$

where *y*(*t*) represents VO_2_ as a function of time during the transition to the steady state of new PO. *y*_*BSL*_ is VO_2_ before the transition, *A*_*p*_ is the amplitude (increase above *y*_*BSL*_), *t* is the dependent time variable, *TD* is the time delay, and *τ* is the time constant (time that elapses for 63% of the response to occur). The curve was fit to the data by applying the Levenberg–Marquardt algorithm for non-linear least squares analysis (Origin 9.7; OriginLab, Northampton, MA, United States).

### Statistics

Ten participants were recruited based on a sample size power calculation of the measured VO_2max_. It was found that 10 participants were sufficient (80% power) to calculate pre vs. post VO_2max_ (α = 0.05) to within an SD of ± 30 mL with the consideration of a 20% dropout rate. Mean analyses from 0 s (start of square-wave change in PO) to 360 s (end of exercise) (*n* = 10) of VO_2_, VCO_2_, P_ET_O_2_, P_ET_CO_2_, V_E_, f_B_, VTI, Δ[HHb], Δ[Hb_tot_], S_at_O_2_, and Δ[HHb]/VO_2_ between conditions (CONLD, CONLD-BH, FLK, and FLK-BH) were performed by a one-way repeated measures (1-RM) analysis of variance (ANOVA). [La^–^] was compared between conditions and time (PRE vs. POST) by a two-way RM (2-RM) ANOVA. The first 25 s and last 5 s of each apneic cycle were analyzed for VO_2_ and VCO_2_ *via* a 2-RM ANOVA. The Shapiro–Wilk and Brown–Forsythe tests were performed to assess the normality and heteroscedasticity of the data, respectively. The 1-RM ANOVA comparisons for VCO_2_, P_ET_O_2_, P_ET_CO_2_, V_E_, f_B_, VTI, Δ[HHb], Δ[Hb_tot_], S_at_O_2_, and Δ[HHb]/VO_2_ were performed by a Friedman RM ANOVA on ranks due to a rejection of either or both tests, whereas mean VO_2_ was evaluated with a parametric 1-RM ANOVA. Data are reported as mean ± SD. The statistical significance threshold was *p* < 0.05.

## Results

**FIGURE 1:**
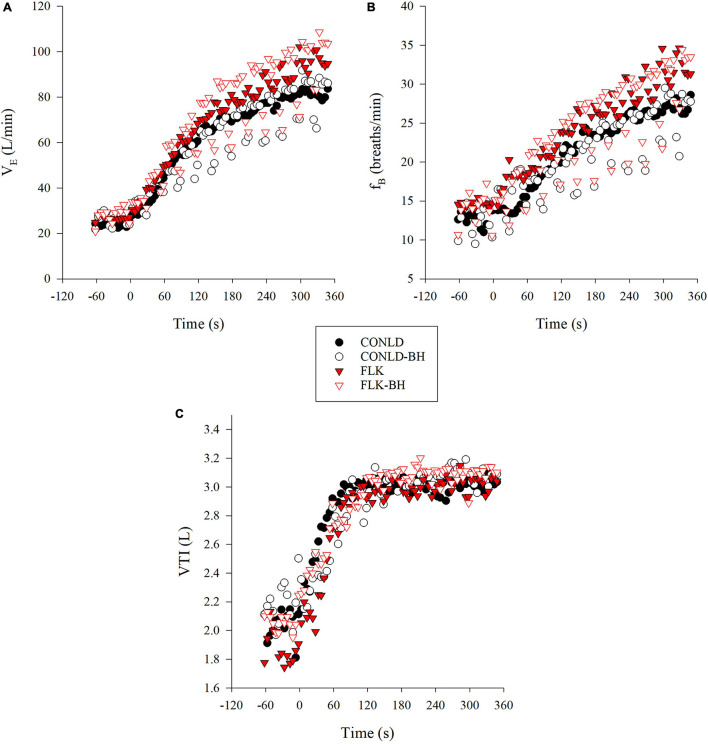
Breathing data during CONLD (filled circles), CONLD-BH (open circles), FLK (filled red triangles), and FLK-BH (open red triangles). **(A)** Minute ventilation (VE), **(B)** frequency of breathing (f^B^), and **(C)** inspiratory tidal volume (VTI). Time 0 marks the start of the exercise transition. Data were interpolated from breath-by-breath to second-by-second and 5 s averaged for graphical representation.

Participants successfully matched V_E_ between CONLD and CONLD-BH (*p* = 0.889) and between FLK and FLK-BH (*p* = 0.889) with regard to the onset of the square-wave increase in PO and exercise cessation (Table 2 and Figure 1A). This was supported by the sustained mean f_B_ between CONLD and CONLD-BH (*p* = 0.72) and between FLK and FLK-BH (*p* = 0.99) (Figure 1B) and mean VTI between CONLD and CONLD-BH (*p* = 0.953) (Figure 1C). VTI was statistically greater in FLK-BH compared to FLK (*p* < 0.001) by ∼80 mL/min.

**TABLE 2:** Breathing frequency (f_B_), inspiratory tidal volume (VTI), and minute ventilation (V_E_) under each condition.

Variable	CONLD	CONLD-BH	FLK	FLK-BH
f_B_ (breaths/min)	22.0 ± 4.7	21.2 ± 4.5	25.2 ± 5.2^a,b^	24.2 ± 6.0^a,b^
VTI (L)	2.93 ± 0.21	2.91 ± 0.27	2.86 ± 0.30^a,b^	2.94 ± 0.24^a,c^
V_E_ (L/min)	64.8 ± 16.9	62.4 ± 17.5	72.7 ± 20.0^a,b^	73.1 ± 22.1^a,b^

*SD, standard deviation. CONLD-BH was matched to CONLD and FLK-BH to FLK. Values are mean ± SD.*

*^a^Significantly different than CONLD.*

*^b^Significantly different than CONLD-BH.*

*^c^Significantly different than FLK.*

### Gas Exchange Variables

**FIGURE 2:**
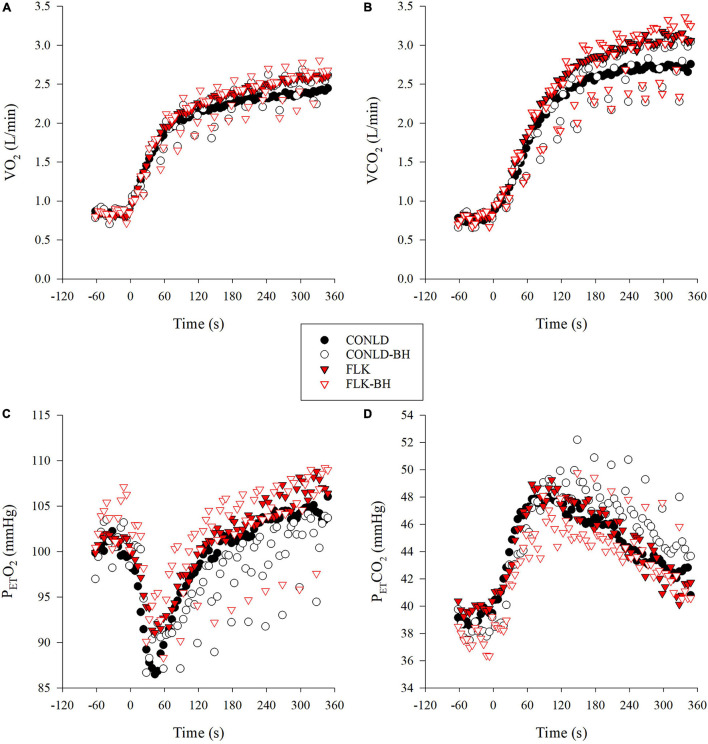
Mean participant respiratory variables during constant A20% work (CONLD, filled circles), constant A20% work with 5 s BHs every 30 s (CONLD-BH, open circles), ?20% work with 5 s sprints at peak power output (PPO) every 30 s (FLK, filled red triangles) and A20% work with 5 s BHs, and sprints at PPO every 30 s (FLK-BH, open red triangles) for 6 min. **(A)** Oxygen consumption (VO_2_), **(B)** carbon dioxide elimination (VCO_2_), **(C)** end-tidal partial pressure of oxygen (PETO_2_), and **(D)** end-tidal partial pressure of carbon dioxide (PETCO_2_). Time 0 marks the start of the exercise transition. Data were interpolated from breath-by-breath to second-by-second and 5 s averaged for graphical representation.

Mean VO_2_ from time 0 to 360 s was similar between CONLD and CONLD-BH (*p* = 0.406) and between FLK and FLK-BH (*p* = 0.165) (Table 3 and Figure 2A). VO_2_ was greater in FLK and FLK-BH than in CONLD (*p* < 0.001 for both comparisons) and greater in FLK than CONLD-BH (*p* < 0.001) over the same time period. Mean VO_2_ for the last 5 s of each 30 s cycle was greater in CONLD than CONLD-BH (*p* = 0.001) and greater in FLK than in FLK-BH (*p* < 0.001) and CONLD-BH (*p* < 0.001) (Table 4). Mean P_ET_O_2_ was greater in CONLD than CONLD-BH (*p* < 0.001) but was unchanged in FLK compared to FLK-BH (*p* = 0.682) (Table 3 and Figure 2C). P_ET_O_2_ was lower in CONLD and CONLD-BH than in FLK and FLK-BH (*p* < 0.001 for all comparisons) (Table 3 and Figure 2C). P_ET_CO_2_ was lower in CONLD than CONLD-BH (*p* < 0.001) but was greater in FLK than FLK-BH (*p* < 0.001) (Table 3 and Figure 2D). VCO_2_ from 0 s to the end of exercise was similar between CONLD and CONLD-BH (*p* = 0.914) and between FLK and FLK-BH (*p* = 0.641), but was greater in FLK and FLK-BH than in CONLD and CONLD-BH (*p* < 0.001 for all comparisons) (Table 3 and Figure 2B).

**TABLE 3:** Mean outcome measures for each of the four conditions, namely, CONLD, CONLD-BH, FLK, and FLK-BH from 0 to 360 s.

Variable	CONLD	CONLD-BH	FLK	FLK-BH
VO_2_ (L/min)	2.12 ± 0.35	2.15 ± 0.42	2.24 ± 0.40^a,b^	2.20 ± 0.45^a^
VCO_2_ (L/min)	2.30 ± 0.55	2.29 ± 0.64	2.55 ± 0.65^a,b^	2.43 ± 0.70^a,b^
P_ET_O_2_ (mmHg)	99.4 ± 5.3	96.9 ± 4.9^a^	101.3 ± 5.0^a,b^	101.5 ± 5.4^a,b^
P_ET_CO_2_ (mmHg)	45.0 ± 2.2	46.3 ± 3.0^a^	45.1 ± 2.6^b^	44.0 ± 2.6^a,b,c^
Δ[Hb_tot_] (μM)	3.3 ± 1.6	-2.5 ± 1.2^a^	2.0 ± 1.6^a,b^	0.82 ± 1.4^a,b,c^
Δ[HHb] (μM)	7.3 ± 1.8	7.0 ± 2.0^a^	6.7 ± 1.8^a,b^	8.7 ± 2.4^a,b,c^
S_at_O_2_ (%)	62.6 ± 1.9	59.4 ± 3.3^a^	61.2 ± 2.2^a,b^	61.7 ± 3.1^a,b^
Δ[HHb]/VO_2_	3.38 ± 0.52	3.25 ± 0.83	2.96 ± 0.64^a,b^	3.88 ± 0.98^a,b,c^
POST [La^–^] (mM)	9.4 ± 2.4	10.0 ± 2.6	11.5 ± 2.5^a,b^	11.3 ± 2.8^a,b^

*SD, standard deviation. Data are reported as mean ± SD.*

*^a^Significantly different than CONLD.*

*^b^Significantly different than CONLD-BH.*

*^c^Significantly different than FLK. POST is 3 min post-exercise.*

**TABLE 4:** Mean outcome measures for both BH conditions (CONLD-BH and FLK-BH) for each 30 s apneic cycle (25 s regulated breathing, 5 s apnea) from 0 to 360 s.

Variable	CONLD-BH		FLK-BH x	
	25 s	5 s	25 s	5 s
VO_2_ (L/min)	2.19 ± 0.24	1.93 ± 0.27*	2.25 ± 0.38	1.89 ± 0.27*
VCO_2_ (L/min)	2.33 ± 0.23	2.04 ± 0.27*	2.49 ± 0.34	2.11 ± 0.22*
P_ET_O_2_ (mmHg)	98.0 ± 4.4	90.9 ± 3.7*	103.0 ± 4.9	93.5 ± 4.4*
P_ET_CO_2_ (mmHg)	45.8 ± 4.1	48.8 ± 4.1*	43.4 ± 4.6	47.2 ± 4.3*
Δ[HHb] (μM)	7.0 ± 6.2	7.3 ± 6.5*	8.6 ± 9.6	8.9 ± 9.8*

*SD, standard deviation. Data are reported as mean ± SD.*

*25 s significantly different than 5 s.

### Δ[Hb_tot_], Δ[HHb], S_at_O_2_, and Δ[HHb]/VO_2_

**FIGURE 3:**
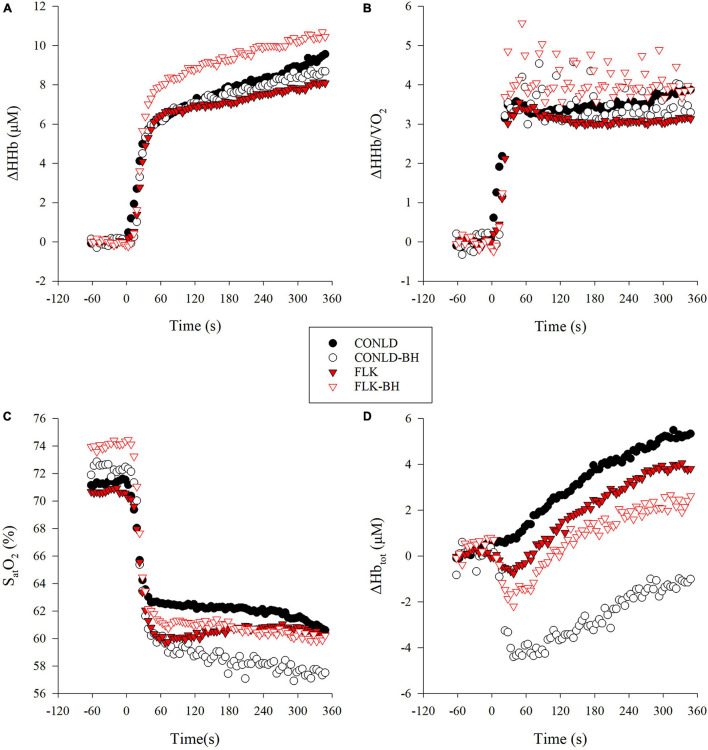
Local muscle deoxygenation variables from near infrared spectrometry (NIRS) data in CONLD (filled circles), CONLD-BH (open circles), FLK (filled red triangles), and FLK-BH (open red triangles). **(A)** Deoxyhemoglobin concentration normalized to baseline (Δ[HHb]), **(B)** ratio of normalized deoxyhemoglobin to VO_2_ (Δ[HHb]/VO_2_), **(C)** oxygen saturation of hemoglobin S_at_O_2_, and **(D)** total hemoglobin concentration normalized to baseline (Δ[Hb_tot_]). Time 0 marks the start of the exercise transition. Data were interpolated from breath-by-breath to second-by-second and 5 s averaged for graphical representation.

NIRS-derived normalized total hemoglobin content (Δ[Hb_tot_]) was greater in CONLD compared to CONLD-BH (*p* < 0.001), FLK (*p* < 0.001), and FLK-BH (*p* < 0.001), and also in FLK compared to FLK-BH (*p* < 0.001) (Table 3 and Figure 3C). Deoxygenated hemoglobin normalized to baseline (Δ[HHb]) was greater in CONLD than in CONLD-BH (*p* = 0.011) and FLK (*p* < 0.001), and also in FLK-BH than in other three conditions (*p* < 0.001 for all comparisons) from the onset to the cessation of exercise (Table 3 and Figure 3A). S_at_O_2_ was greater in CONLD compared to CONLD-BH (*p* < 0.001), FLK (*p* < 0.001), and FLK-BH (*p* < 0.001), and it was not significantly different in FLK compared to FLK-BH (*p* = 0.434) (Table 3 and Figure 3D). The ratio of Δ[HHb]/VO_2_ was similar between CONLD and CONLD-BH (*p* = 0.086), but lower in FLK than FLK-BH (*p* < 0.001) (Table 3 and Figure 3B).

### Lactate ([La^–^])

**FIGURE 4:**
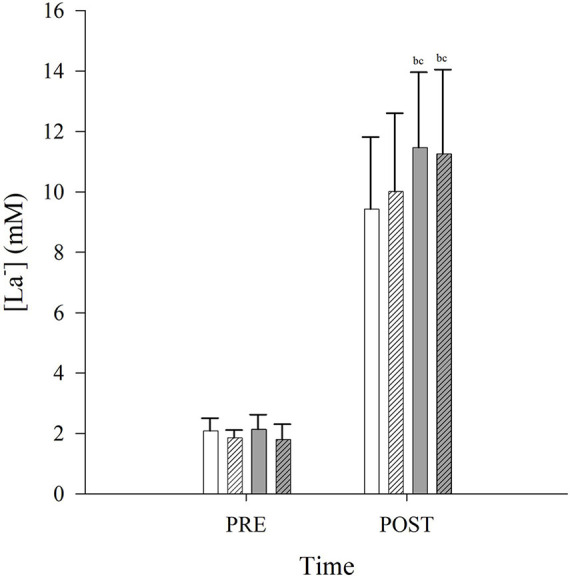
Arterialized capillary lactate concentrations ([La^–^]) PRE and POST exercise in CONLD (white), CONLD-BH (white with diagonal lines), FLK (gray) and FLK-BH (gray with diagonal lines). All four POST [La^–^] were greater than the PRE [La^–^]. ^a^Significantly greater than CONLD. ^b^Significantly greater than POST CONLD. ^c^Significantly greater than POST CONLD-BH.

Post-exercise arterialized capillary lactate concentration ([La^–^]) was unchanged in CONLD compared to CONLD-BH (*p* = 1.000), and both were lower compared to FLK and FLK-BH (*p* < 0.001 and *p* = 0.007, respectively) (Table 3 and Figure 4). Post-exercise [La^–^] was similar between FLK and FLK-BH (*p* = 1.000) (Table 3 and Figure 4).

## Discussion

The goal of this study was to monitor the physiological adjustments to repeated cycles of 5 s BHs followed by 25 s of regulated breathing during CONLD and FLK exercise that lasted 6 min. Main findings included (i) unchanged VO_2_ between CONLD and CONLD-BH, and in FLK and FLK-BH, despite the imposed regulatory breathing paradigm; (ii) Δ[Hb_tot_] and S_at_O_2_ were lower, and a marginal decrease in Δ[HHb] was observed in CONLD-BH compared to CONLD, whereas Δ[HHb] and S_at_O_2_ were greater in FLK-BH compared to FLK.

### CONLD and CONLD-BH

The changes in PO in CONLD and CONLD-BH, relative to the previous related research (Δ50%-Δ20%) (Lim et al., 2018), reflect the additions of the regulated breathing to this 25 s breathing/5 s apnea protocol. The purpose of this modification was to simulate the difference in breathing patterns between swimming backstroke (25 s free-breathing) and front crawl (25 s regulated breathing). By introducing these restrictions, participants were forced to perform a much lower intensity (189 W, Δ20%) compared to that suggested by Lim et al. (2018) (218 W, Δ50%, *p* = 0.027), despite having similar aerobic fitness (VO_2max_: 3.23 and 3.17 L/min, *p* = 0.82, respectively), LTs (VO_2_ at LT: 1.72 and 1.77 L/min, *p* = 0.57, respectively; PO at LT: 155 and 156 W, respectively), and PPO (303 and 314 W, *p* = 0.58, respectively) to perform the 6 min trials. Moreover, the reduced PO of this study elicited lower mean V_E_ (68 L/min) compared to that suggested by Lim et al. (99 L/min) (Lim et al., 2018). Within the context of this PO adjustment of this study, VO_2_ was unchanged with the addition of the 5 s apneic periods and matching of f_B_ and VTI during the 25 s breathing periods (Table 2) of CONLD-BH, demonstrating that any potential increase in O_2_ cost derived from the periodic apneas had been met. The data in this study show that the aerobic metabolic demand (i.e., VO_2_) was supported by the decreased S_at_O_2_ (Table 3 and Figure 3C) and increased muscle deoxygenation under the reduced perfusion conditions, as reflected by the much greater decrease in mean Δ[Hb_tot_] (Table 3 and Figure 3D) and modest decrease in Δ[HHb] (Table 3 and Figure 3A). A similar reduction in S_at_O_2_ and an increase in muscle deoxygenation were observed by Kume et al. (2016) at a PO corresponding to 65% of VO_2max_ interspersed with 4 s exhalations followed by a maximal inhalation, simulating hypoventilation. Furthermore, previous work by Hoffmann et al. (2005) under apneic and rebreathing exercise conditions during higher intensity exercise, which generated similar hypoxia (decreased P_ET_O_2_ in rebreathing), demonstrated greater mean arterial pressure and lower heart rate in the apneic compared to the rebreathing condition, demonstrating that only the apneic state elicited the observed O_2_ conservation or diving response that is associated with the decreased intramuscular blood flow. They concluded that the mechanical cessation of breathing was the key stimulus to this drop in perfusion. This has been corroborated by similar protocols comparing rebreathing with apnea (Lindholm et al., 1999). The data of this study confirm a similar response under the combination of the regulated breathing and BH paradigm. Moreover, the observed decrease in Δ[Hb_tot_] shown in this study was not observed during hypoxic exercise (∼12% fraction of oxygen in inspired air [F_I_O_2_]) performed at low-moderate (single-leg knee extensions) (DeLorey et al., 2004) and supra-LT intensities (Ainslie et al., 2007), suggesting again that the reduced blood flow in this study was apnea-related. Furthermore, earlier research by Lim et al. (2018), within a comparable protocol as this study, and Kume et al. (2013), performing intermittent apneas during continuous exercise at a comparable intensity, identified similar temporal reductions in Δ[Hb_tot_] resolved by similar increases in muscle deoxygenation. This decrease was observed despite previous suggestions that Δ[Hb_tot_] is increased during deep diving *via* splenic contractions (Hurford et al., 1990).

The drop in P_ET_O_2_ (Table 4 and Figure 2C) and increased VO_2_ (Table 4 and Figure 2A) observed immediately post-apnea in this study reflect continued alveolar to capillary O_2_ diffusion facilitating the unchanged VO_2_. This response has also been observed under 20 s apneas (Lindholm and Linnarsson, 2002) and also reflected the actual arterial O_2_ (P_a_O_2_) under these transient apneic conditions (Suskind et al., 1950).

The maintenance of mean VO_2_ between CONLD and CONLD-BH after the exercise on-transient response was supported not only by the microvasculature hemodynamic changes outlined earlier, but also by the increased VO_2_ during the 25 s of regulated breathing (Table 4 and Figure 2A) and the reduced P_ET_O_2_ immediately post-BH (Table 4 and Figure 2C) suggested earlier (Yamamoto et al., 1987). Specifically, Woorons et al. (2007) found that intermittent apneas performed at a high pulmonary volume (i.e., near total lung capacity) during higher intensity exercise (∼70% VO_2max_), similar to this study, maintained pulmonary arterial S_at_O_2_.

The unchanged mean VCO_2_, between CONLD and CONLD-BH, suggests that the H^+^ buffering, associated with the lactate production of anaerobic glycolysis, has been reflected in the observed increase in P_ET_CO_2_ and that the BH did not impose a greater anaerobic glycolytic contribution. The unchanged post-exercise [La^–^] between CONLD and CONLD-BH trials is in contrast to other studies that have reported increased [La^–^] accumulation during incremental (Seo et al., 2020) (50 W + 12.5 W/min to exhaustion) and intermittent (Lim et al., 2018) exercise under hypoxic or apneic condition, respectively. However, these studies were performed at much higher supra-LT PO, which would elicit much greater blood [La^–^].

From a swimming front crawl perspective, at this Δ20% PO intensity, the addition of the regulated breathing paradigm and the 5 s BH, while maintaining a similar intensity of kicking, may be performed without negative physiological consequences. Notably, this is within the context of the legs only paradigm of this experiment as opposed to the simultaneous arm and leg action that is performed after the underwater portion of the swim.

### FLK and FLK-BH

The FLK condition mimics the strategies of those swimmers who believe that sprint kicking during the underwater portion after the turn, along with their own superior hydrodynamics compared to their opponents, will give them a competitive advantage. In contrast to the study by Lim et al. (2018), utilizing a similar protocol, albeit at much higher PO, our results showed that implementing the intermittent 5 s periods of apnea to FLK (FLK-BH) did not affect mean VO_2_. Similar to CONLD-BH and CONLD, f_B_ and VTI were also regulated in FLK-BH to FLK; however, although VTI was statistically greater in FLK-BH (Table 2), the unchanged V_E_ suggested that this ∼80 mL/min change in VTI (Δ1.6% over the 6 min) had no physiological effects. It was notable that VTI was lower in FLK compared to CONLD (2.86 ± 0.30 L and 2.93 ± 0.21 L, respectively). However, coupled with the increased f_B_ (25.2 ± 5.2 breaths/min and 22.0 ± 4.7 breaths/min), a higher V_E_, in conjunction with the higher VCO_2_ in FLK vs. CONLD, was observed (Table 2). The lowered P_ET_O_2_ immediately post-apnea (Table 4 and Figure 2A) suggests that the observed pulmonary alveoli to capillary diffusion continued during the 5 s facilitating O_2_ transport. Moreover, the increased Δ[HHb] and Δ[HHb]/VO_2_ in FLK-BH compared to FLK, which reflected a greater reliance on muscle deoxygenation, at the active muscle was responsible for this result (duManoir et al., 2010; Murias et al., 2011).

In elite swimmers, similar maintenance of VO_2_ has been demonstrated during 4 min of submaximal intensity swimming with apnea induced by regulated breathing conditions of every two arm strokes up to a maximum of five-arm strokes, notwithstanding the reduced V_E_ in the latter condition (Dicker et al., 1980). The consequences of the imposed regulated breathing and comparable intensity in the current study were apparently resolved by the greater alveolar to pulmonary capillary O_2_ diffusion. The muscle deoxygenation response was not recorded in the earlier work by Dicker et al. (1980). However, a similar increase in Δ[HHb] was observed by Billaut and Buchheit (2013) during 10 repeated 10 s maximal sprints followed by 30 s rest, under hypoxic conditions (13% F_I_O_2_) compared to normoxia. Conversely, others have studied the effects of repeated 3 s loadless cycling recovery periods, as opposed to the 5 s of high PO as in this study, and observed an improved microvascular O_2_ delivery reflected by decreased Δ[HHb]/VO_2_ (Belfry et al., 2012). It is suggested that the contrasting results of this study were attributed to the apneic O_2_ conservation effect (Kume et al., 2013), as reflected by the decreased Δ[Hb_tot_] in FLK-BH compared to FLK. This result is similar to the CONLD-BH compared to CONLD, of this study and others, reflecting a similar redistribution of blood flow away from working muscles during apnea (Kume et al., 2013; Lim et al., 2018). Furthermore, it is suggested that the unchanged mean VCO_2_ and [La^–^] during FLK-BH compared to FLK despite the apneic periods was a consequence of the continued buffering and pulmonary capillary to alveolar diffusion and buffering during this 5 s BH (Table 4). Conversely, Lim et al. (2018), under a similar apnea protocol (5 s), but with free-breathing (25 s), as opposed to regulated breathing of this study, showed lower mean VCO_2_ in FLK-BH compared to FLK and higher lactates associated with the higher mean PO (Δ50% vs. Δ20%), suggesting a relative decrease in ventilatory buffering at this much higher relative PO.

To our knowledge, this was the first study that regulated breathing during supra-threshold PO exercise on a cycle ergometer following periods of apnea, to a free-breathing protocol at the same PO similar to the lower extremity work associated with swimming in the prone position. Consequently, mean VO_2_ was maintained in these apneic conditions (CONLD-BH and FLK-BH) compared to their free-breathing counterparts (CONLD and FLK, respectively) by greater muscle deoxygenation, despite decreased intramuscular blood perfusion. The regulated f_B_ and V_E_ during the 25 s breathing periods of this study eliminated the transient increases in V_E_ that previously corresponded with greater post-apneic VO_2_ and VCO_2_ (Lim et al., 2018). VO_2_ was sustained in CONLD-BH compared to CONLD, and in FLK-BH compared to FLK, as a function of the continued pulmonary diffusion during apnea, as is reflected by the immediate increases in P_ET_O_2_ post-apnea (Table 4). At the level of the muscle, the preserved arterial O_2_ content along with the increased muscle deoxygenation facilitated the unchanged VO_2_. Although these protocols were designed to replicate the breathing opportunities afforded during regulated compared to free-breathing conditions, the cardiorespiratory responses to facial immersion supine exercise, and the combined arm and leg muscle action specific to swimming (Guyatt et al., 1965; Christie et al., 1990; Leahy et al., 2019) were not studied, due to the inherent difficulties of calculating gas exchange while submerged, and the unavailability of a recumbent ergometer that would interface successfully with our data collection equipment. Additional research is needed to clarify the role of these specific swimming characteristics in these physiological outcomes. Furthermore, our results may not reflect the responses of well-trained competitive swimmer, and as such, future studies should compare the physiological resolution of expert swimmers, compared to non-swimmers, to these breathing restrictions. Finally, only healthy participants were tested; therefore, no comparisons to diseased populations should be made.

Moreover, only male participants were included in this study to best match participant characteristics to the previous study (Lim et al., 2018). Therefore, these observations may not apply to females because of sex differences in body composition, fluctuations in reproductive hormones (estrogen and progesterone) (Arora et al., 1998), and lactate production during exercise (Jurkowski et al., 1981). Also, the sample size (*n* = 10) of this study adequately powered the statistical analyses used; a larger sample size may be required to generalize these results.

## Conclusion

The initial and necessary reduction of PO (Δ50%–Δ20%) imposed by the regulated breathing condition demonstrated the severe cardiorespiratory consequences of this regulated breathing protocol compared to the free-breathing paradigm instituted previously by our lab. However, under this reduced PO, mean VO_2_ was maintained after the implementation of 5 s apneic periods and 25 s regulated post-BH breathing during supra-threshold exercise. The mechanism for this sustained VO_2_ under the apnea condition, with its reduced breathing opportunities, was expounded through an increase in muscle deoxygenation (Δ[HHb]) relative to VO_2_ within the constraints of the O_2_ conservation or deep diving response (decreased Δ[Hb_tot_]). This was in contrast with the systematic increases in V_E_ and unchanged Δ[HHb]/VO_2_ observed during free as opposed to regulated breathing conditions, under an otherwise identical apneic protocol compared to this study. From a practical perspective, swimmers competing in the predominantly aerobic front crawl events (400, 800, and 1,500 m) would be advised to increase their minute ventilation by increasing the frequency of breathing to twice per arm cycle as often as is comfortable, increase tidal volumes, or suffer negative performance consequences.

## Data Availability Statement

The raw data supporting the conclusions of this article will be made available by the authors, without undue reservation.

## Ethics Statement

The studies involving human participants were reviewed and approved by the Western University Research Ethics Board for Health Sciences Research Involving Human Participants. The patients/participants provided their written informed consent to participate in this study.

## Author Contributions

GB and DL conceived, designed the study, collected, and analyzed the data. GB and KG interpreted the results and drafted the manuscript. GB and JM edited the manuscript. All authors contributed to the article and approved the submitted version.

## Conflict of Interest

The authors declare that the research was conducted in the absence of any commercial or financial relationships that could be construed as a potential conflict of interest.

## Publisher’s Note

All claims expressed in this article are solely those of the authors and do not necessarily represent those of their affiliated organizations, or those of the publisher, the editors and the reviewers. Any product that may be evaluated in this article, or claim that may be made by its manufacturer, is not guaranteed or endorsed by the publisher.

## References

[B1] Ainslie P. N. Barach A. Murrell C. Hamlin M. Hellemans J. Ogoh S. (2007). Alterations in cerebral autoregulation and cerebral blood flow velocity during acute hypoxia: rest and exercise. *Am. J. Physiol. Heart Circul. Physiol.* 292 H976–H983. 10.1152/ajpheart.00639.2006 17012355

[B2] Andersson J. P. A. Linér M. H. Fredsted A. Schagatay E. K. A. (2004). Cardiovascular and respiratory responses to apneas with and without face immersion in exercising humans. *J. Appl. Physiol.* 96 1005–1010. 10.1152/japplphysiol.01057.2002 14578373

[B3] Arora S. Veves A. Caballaro A. E. Smakowski P. LoGerfo F. W. (1998). Estrogen improves endothelial function. *J. Vasc. Surg.* 27 1141–1147. 10.1016/S0741-5214(98)70016-3 9652476

[B4] Babcock M. A. Paterson D. H. Cunningham D. A. Dickinson J. R. (1994). Exercise on-transient gas exchange kinetics are slowed as a function of age. *Med. Sci. Sports Exer.* 26 440–446. 8201899

[B5] Beaver W. L. Wasserman K. Whipp B. J. (1986). A new method for detecting anaerobic threshold by gas exchange. *J. Appl. Physiol.* 60 2020–2027. 10.1152/jappl.1986.60.6.2020 3087938

[B6] Belfry G. R. Paterson D. H. Murias J. M. Thomas S. G. (2012). The effects of short recovery duration on VO2 and muscle deoxygenation during intermittent exercise. *Eur. J. Appl. Physiol.* 112 1907–1915. 10.1007/s00421-011-2152-4 21927832

[B7] Billaut F. Buchheit M. (2013). Repeated-sprint performance and vastus lateralis oxygenation: effect of limited O2 availability. *Scand. J. Med. Sci. Sports* 23 e185–93. 10.1111/sms.12052 23362832

[B8] Chacaroun S. Vega-Escamilla y Gonzalez I. Flore P. Doutreleau S. Verges S. (2019). Physiological responses to hypoxic constant-load and high-intensity interval exercise sessions in healthy subjects. *Eur. J. Appl. Physiol.* 119 123–134. 10.1007/s00421-018-4006-9 30315366

[B9] Christie J. L. Sheldahl L. M. Tristani F. E. Wann L. S. Sagar K. B. Levandoski S. G. (1990). Cardiovascular regulation during head-out water immersion exercise. *J. Appl. Physiol.* 69 657–664. 10.1152/jappl.1990.69.2.657 2228879

[B10] DeLorey D. S. Shaw C. N. Shoemaker J. K. Kowalchuk J. M. Paterson D. H. (2004). The effect of hypoxia on pulmonary O2 uptake, leg blood flow and muscle deoxygenation during single-leg knee-extension exercise. *Exp. Physiol.* 89 293–302. 10.1113/expphysiol.2003.026864 15123565

[B11] Dicker S. G. Lofthus G. K. Thornton N. W. Brooks G. A. (1980). Respiratory and heart rate responses to tethered controlled frequency breathing swimming. *Med. Sci. Sports Exerc.* 12 20–23. 7392897

[B12] duManoir G. R. DeLorey D. S. Kowalchuk J. M. Paterson D. H. (2010). Kinetics of VO2 limb blood flow and regional muscle deoxygenation in young adults during moderate intensity, knee-extension exercise. *Eur. J. Appl. Physiol.* 108 607–617. 10.1007/s00421-009-1263-7 19882164

[B13] Ferrigno M. Ferretti G. Ellis A. Warkander D. Costa M. Cerretelli P. (1997). Cardiovascular changes during deep breath-hold dives in a pressure chamber. *J. Appl. Physiol.* 83 1282–1290. 10.1152/jappl.1997.83.4.1282 9338438

[B14] Guyatt A. R. Newman F. Cinkotai F. F. Palmer J. I. Thomson M. L. (1965). Pulmonary diffusing capacity in man during immersion in water. *J. Appl. Physiol.* 20 878–881. 10.1152/jappl.1965.20.5.878 5837613

[B15] Hoffmann U. Smerecnik M. Leyk D. Essfeld D. (2005). Cardiovascular responses to apnea during dynamic exercise. *Int. J. Sports Med.* 26 426–431. 16037883 10.1055/s-2004-821113

[B16] Hurford W. E. Hong S. K. Park Y. S. Ahn D. W. Shiraki K. Mohri M. (1990). Splenic contraction during breath-hold diving in the Korean ama. *J. Appl. Physiol.* 69 932–936. 10.1152/jappl.1990.69.3.932 2246181

[B17] Inglis E. C. Iannetta D. Murias J. M. (2017). The plateau in the NIRS-derived [HHb] signal near the end of a ramp incremental test does not indicate the upper limit of O2 extraction in the vastus lateralis. *Am. J. Physiol. Regul. Integr. Compar. Physiol.* 313 R723–R729. 10.1152/ajpregu.00261.2017 28931547 PMC5814694

[B18] Jurkowski J. Jones N. L. Toews C. J. Sutton J. R. (1981). Effects of menstrual cycle on blood lactate, O2 delivery, and performance during exercise. *J. Appl. Physiol.* 51 1493–1499. 6798000 10.1152/jappl.1981.51.6.1493

[B19] Keir D. A. Murias J. M. Paterson D. H. Kowalchuk J. M. (2014). Breath-by-breath pulmonary O2 uptake kinetics: effect of data processing on confidence in estimating model parameters. *Exp. Physiol.* 99 1511–1522. 10.1113/expphysiol.2014.080812 25063837

[B20] Kennedy M. D. Warburton D. E. R. Boliek C. A. Esch B. T. A. Scott J. M. Haykowsky M. J. (2008). The oxygen delivery response to acute hypoxia during incremental knee extension exercise differs in active and trained males. *Dyn. Med.* 7:11. 10.1186/1476-5918-7-11 18700024 PMC2526084

[B21] Kume D. Akahoshi S. Song J. Yamagata T. Wakimoto T. Nagao M. (2013). Intermittent breath holding during moderate bicycle exercise provokes consistent changes in muscle oxygenation and greater blood lactate response. *J. Sports Med. Phys. Fitness* 53 327–335. 23715258

[B22] Kume D. Akahoshi S. Yamagata T. Wakimoto T. Nagao N. (2016). Does voluntary hypoventilation during exercise impact EMG activity? *Springerplus* 5:149. 10.1186/s40064-016-1845-x 27026846 PMC4766162

[B23] Lamarra N. Whipp B. J. Ward S. A. Wasserman K. (1987). Effect of interbreath fluctuations on characterizing exercise gas exchange kinetics. *J. Appl. Physiol.* 62 2003–2012. 10.1152/jappl.1987.62.5.2003 3110126

[B24] Leahy M. G. Summers M. N. Peters C. M. Molgat-Seon Y. Geary C. M. Sheel A. W. (2019). The mechanics of breathing during swimming. *Med. Sci. Sports Exerc.* 51 1467–1476. 30649105 10.1249/MSS.0000000000001902

[B25] Lim D. J. Kim J. J. Marsh G. D. Belfry G. R. (2018). Physiological resolution of periodic breath holding during heavy-intensity Fartlek exercise. *Eur. J. Appl. Physiol.* 118 2627–2639. 10.1007/s00421-018-3986-9 30206692

[B26] Lindholm P. Linnarsson D. (2002). Pulmonary gas exchange during apnoea in exercising men. *Eur. J. Appl. Physiol.* 86 487–491. 10.1007/s00421-002-0581-9 11944095

[B27] Lindholm P. Sundblad P. Linnarsson D. (1999). Oxygen-conserving effects of apnea in exercising men. *J. Appl. Physiol.* 87 2122–2127. 10.1152/jappl.1999.87.6.2122 10601158

[B28] Murias J. M. Spencer M. D. DeLorey D. S. Gurd B. J. Kowalchuk J. M. Paterson D. H. (2011). Speeding of V̇O-2 kinetics during moderate-intensity exercise subsequent to heavy-intensity exercise is associated with improved local O2 distribution. *J. Appl. Physiol.* 111 1410–1415. 10.1152/japplphysiol.00607.2011 21836042

[B29] Seo J.-B. Kim S.-W. Jung W.-S. Park H.-Y. Lim K. (2020). Effects of various hypobaric hypoxia on metabolic response, skeletal muscle oxygenation, and exercise performance in healthy males. *J. Mens Health* 16 107–120. 10.31083/jomh.v16i4.312 31345004

[B30] Suskind M. Bruce R. A. McDowell M. E. Yu P. N. G. Frank W. Lovejoy J. (1950). Normal variations in end-tidal air and arterial blood carbon dioxide and oxygen tensions during moderate exercise. *J. Appl. Physiol.* 3 282–290. 10.1152/jappl.1950.3.5.282 14794590

[B31] Whipp B. J. Davis J. A. (1979). Peripheral chemoreceptors and exercise hyperpnea. *Med. Sci. Sports* 11 204–212. 40092

[B32] Woorons X. Mollard P. Pichon A. Duvallet A. Richalet J.-P. Lamberto C. (2007). Prolonged expiration down to residual volume leads to severe arterial hypoxemia in athletes during submaximal exercise. *Respirat. Physiol. Neurobiol.* 158 75–82. 10.1016/j.resp.2007.02.017 17434347

[B33] Yamamoto Y. Mutoh Y. Kobayashi H. Miyashita M. (1987). Effects of reduced frequency breathing on arterial hypoxemia during exercise. *Eur. J. Appl. Physiol. Occup. Physiol.* 56 522–527. 10.1007/BF00635364 3653092

